# Interdisciplinary Treatment of Macroglossia Due to a Microcystic Lymphatic Malformation with Bleomycin Electrosclerotherapy Followed by Partial Resection

**DOI:** 10.1007/s00270-024-03693-1

**Published:** 2024-04-30

**Authors:** J. H. Loeser, U. Kisser, L. Dießel, S. von der Heydt, O. Bidakov, C. Loberg, W. A. Wohlgemuth

**Affiliations:** 1 Clinic and Polyclinic of Radiology, University Hospital Halle (Saale), Ernst-Grube-Straße 40, 06120 Halle Saale, Germany; 2 Clinic and Polyclinic of ENT, University Hospital Halle (Saale), Ernst-Grube-Straße 40, 06120 Halle Saale, Germany; 3 Clinic and Polyclinic of Pathology, University Hospital Halle (Saale), Ernst-Grube-Straße 40, 06120 Halle Saale, Germany

Lymphatic malformations are rare congenital vascular anomalies. Treatment remains challenging due to their varying localization and their different morphology especially if located on the tongue. In addition to the typical symptoms, such as pain and recurrent infections, small lymphatic blebs on the surface of the tongue, macroglossia is a particular problem due to accompanying localized soft tissue hyperplasia making mouth closure und eating difficult. In addition, it may be difficult to reach the dorsal and basal parts for the lesion for treatment via direct puncture [1]. The current state-of-the-art treatment of lymphatic malformations is intralesional injection of a sclerosing agent. A new form of therapy is the combination of bleomycin injections followed by reversible electroporation. The application of short electric pulses temporarily increases the cell permeability, allowing for a higher intracellular bleomycin concentration and thus to use lower bleomycin doses overall, as well as reducing the risk of possible side effects. First studies have proven the effectiveness of this new form of therapy applied to different kinds of malformations [2, 3].

A 2-year-old male was admitted to our department with macroglossia, no mouth closure possible and dry-brownish coating parts of the protruding proportion of the tongue with previous partial resection followed by four sclerotherapies with alcohol. Ingestion of food and drinking was significantly impaired while patient’s condition progressively worsened. No other cases in his family were reported (Fig. 1).

**Fig. 1:**
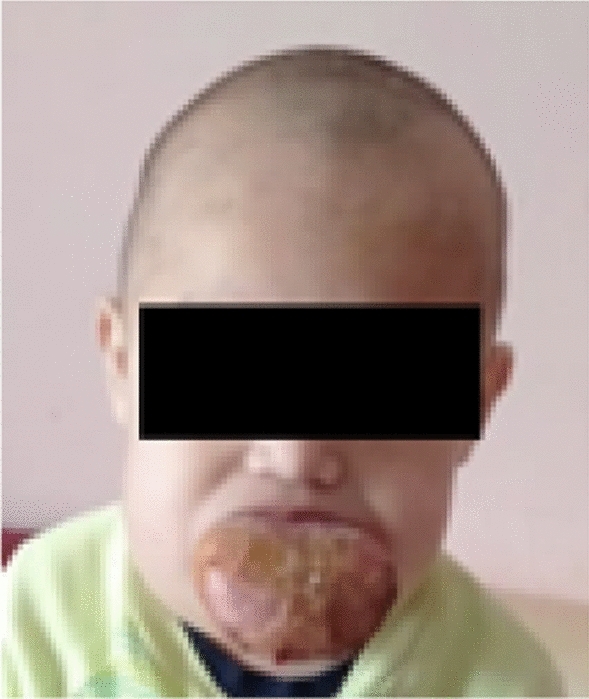
Image of the patient before the course of treatment

A 3 T MRI was performed, which confirmed the diagnosis of a microcystic lymphatic malformation of the entire tongue with cervical extension on both sides. Venous outflow obstruction was excluded by ultrasound examination.

Due to the extent of the malformation, a therapy with the mTOR-inhibitor sirolimus was started in addition to the invasive treatments. Bleomycin electrosclerotherapy (BEST) was performed under general anesthesia. Via direct puncture, a diluted solution of bleomycin (1 mg) and contrast medium was injected into the LM puncture under angiographic control. Using a finger electrode with Clinicporator Vitae (IGEA S.p.A., Italy) reversible electroporation was performed with an interval of a few seconds between each electroporation. After significant improvement of the symptoms as well as size reduction after each BEST treatment, this was repeated two more times within two years on other now accessible areas of the tongue (BEST No. 2: 2 mg; BEST No. 3: 2 mg). In order to further improve lingual morphology and function, we decided on a reduction glossoplasty, now be technically possible after near occlusion of the LM.

The procedure was conducted under general anesthesia while full thickness excision was performed in a keyhole fashion on the dorsum of the tongue and a “W”-shape on the underside in order to preserve the frenulum [4].

Histology revealed mucosal sections covered by a multilayered acanthotic squamous epithelium with superficial compact parakeratosis. The subepithelial connective tissue and adjacent skeletal muscle contained numerous irregular, thin-walled, ectatic vascular structures with an inconspicuous endothelial lining. The vessels partially reach directly subepithelial with consecutive clumsy swelling of the mucosal papillae. Immunohistochemically, the endothelium showed positive reaction with antibodies against D2-40 and CD31, congruent with a lymphatic phenotype (Fig. 2).

**Fig. 2:**
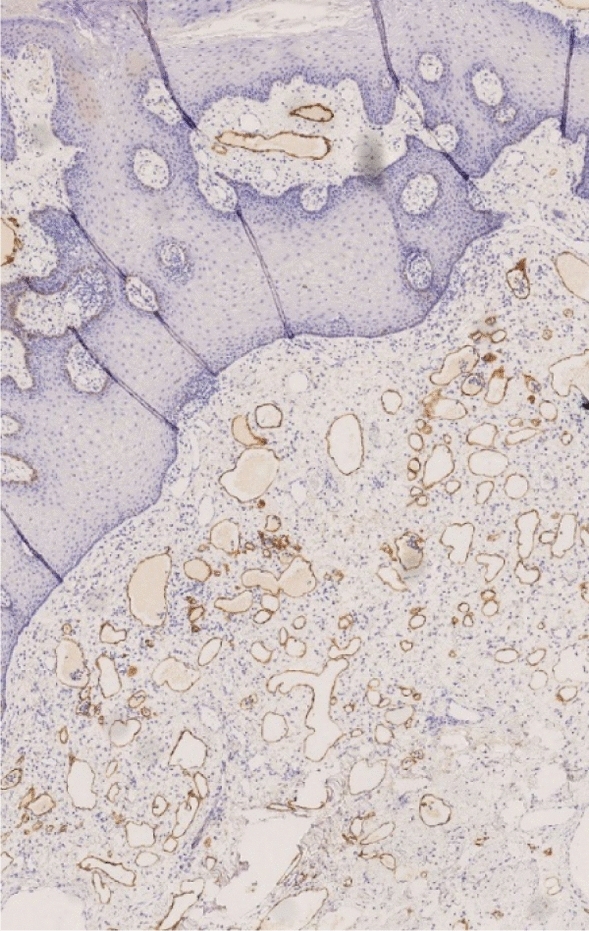
Immunohistochemically findings: D2-40, 40 × Those vessels with positive immune response with D2-40, corresponding to a lymphoid phenotype

Compared to the patient’s initial presentation, he now reports with no symptoms at three months post-surgery. Wounds had healed without complications; the patient can eat almost without any restrictions and can communicate well with still slightly unclear speech. A complete mouth closure is also possible when performed intentionally so that we consider the therapy to be finished for now. A subsequent logopedic treatment could be needed due to years of not uncommon swelling of the LM and the pressure it exerted on the surrounding tissues that led to a dysplastic jaw (Fig. 3).

**Fig. 3:**
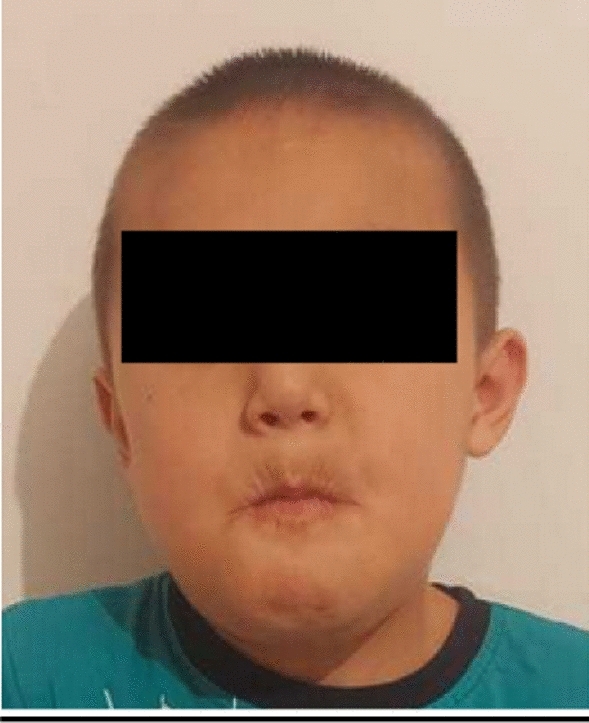
Image of the patient seven months after treatment

In the case described here, it was possible to achieve a considerable volume reduction. A primary wound healing was observed, which is rarely possible with the use of normal sclerotherapy or even resection alone, as well as a reduction of the lymphatic components by BEST. However, the measured volumes and reductions are only an approximation due to the young age of the patient with continuous growth, while it is likely that the reductions could be greater than the values reported here despite the fact that the patient was still at a child growth. The combination of intralesional bleomycin injection and reversible electroporation not only demonstrated a clear sclerosing effect on the malformation but also only low doses of bleomycin were required compared to the usual area of application in chemotherapy. Despite multiple interventions, the cumulative dose of bleomycin of 5 mg remained comparatively low and far below the threshold for systemic lung toxicity. No signs of pulmonary complications attributable to bleomycin and no major complications as defined by the CIRSE classification occurred during the complete course.

By combining the new therapeutic procedure of BEST with subsequent reduction glossoplasty, a significant improvement in lingual morphology and tongue function was achieved. Further studies will be needed to systematically analyze the long-term effect of the treatment strategy described here.

## Data Availability

The datasets analyzed during the current study are not publicly available [Due to the legal medical confidentiality of the treating physicians, a publication of the patient data would violate it and lead to a de-anonymization of the patient] but are available from the corresponding author on reasonable request.

## Abbreviations

BEST: Bleomycin electrosclerotherapy
mTOR- inhibitor: Mammalian target of rapamycin- inhibitor
